# Attitudes and Knowledge Levels of Optometry Students and Educators Towards Artificial Intelligence in Optometric Practice: An Online Cross-Sectional Survey

**DOI:** 10.1007/s44402-026-00053-z

**Published:** 2026-03-06

**Authors:** Fiona Buckmaster, Diane van Staden, Lauren Coetzee

**Affiliations:** 1 University of Huddersfield, Huddersfield, UK; 2 University of KwaZulu-Natal, Durban, South Africa

**Keywords:** Artificial intelligence, Attitude of health personnel, Optometry, Optometry education, Surveys and questionnaires

## Abstract

**Purpose:**

Artificial intelligence (AI) is poised to revolutionise all aspects of eye care practice in the coming decades. However, not much is known about optometry students’ and educators’ attitudes or knowledge levels about AI in optometry.

**Methods:**

A cross-sectional online survey was distributed via Qualtrics to optometry students and educators at all optometry universities in the UK through university optometry student societies, word of mouth, social media and the professional networks of the researchers. The survey explored knowledge levels and training experience in AI, competence levels using AI in a clinical scenario, attitudes towards the utilisation of AI in optometry and AI topics to potentially include in an optometry curriculum.

**Results:**

A total of 254 responses (213 students and 41 educators) were received from 14 out of 15 optometry universities in the UK. Notably, 96% of students had not received any training in AI, and 76% of students reported little or no knowledge about AI applications in optometry. Educators were more likely to have undergone AI training (34%) and had higher knowledge levels than students, with 59% of educators reporting at least some AI knowledge. Across all respondents, those who had undergone AI training had more positive attitudes towards AI in optometry. Educators felt strongly that teaching about AI should be included in the optometry curriculum. Potential AI topics for inclusion in a curriculum were explored. A broad range of topics were identified as important to include; however, ‘how AI tools are developed, trained and validated’ was seen as the least important to teach to optometry students.

**Conclusion:**

Optometry students’ current self-reported knowledge levels about AI applications in optometry are very limited. This research provides an early exploration of the considerations for creating future AI educational initiatives in optometry programmes. However, further research in this area is still required.

Key Points

- Few UK optometry students have received training in artificial intelligence.
- Optometry students were more likely to hold negative attitudes towards artificial intelligence in optometric practice than educators, and had limited self-reported knowledge levels about artificial intelligence applications in optometry.
- Artificial intelligence training was associated with higher self-reported knowledge levels about artificial intelligence in optometry and greater self-reported competence using artificial intelligence tools in optometric practice, indicating a need for universities to begin integrating clinical artificial intelligence education into optometry programmes.

## Introduction

Artificial intelligence (AI) is poised to revolutionise eye care service delivery in the coming decades [1], alongside advances in other healthcare disciplines. AI tools are expected to impact all aspects of optometric practice from alleviating administrative burdens to enabling earlier detection of ocular conditions, enhancing clinical decision-making and improving patient management [2–8]. While there is some evidence to suggest that the utilisation of AI tools may be more widespread in eye care than other health specialities [9], there are still many challenges that must be overcome in order for the benefits of AI to be realised fully in optometric practice [10].

One such challenge is ensuring that the optometric workforce has the appropriate knowledge and training to use AI-powered tools safely and effectively. Long and Magerko [11] defined AI literacy as ‘*a set of competencies that enables individuals to critically evaluate AI technologies; communicate and collaborate effectively with AI; and use AI as a tool online, at home and in the workplace*’ [11, p. 2]. Universities offering optometry programmes should adapt to technological advancements in eye care by integrating AI literacy into their curricula. However, although some healthcare disciplines, such as medicine and dentistry, have begun teaching about AI to their students, a significant gap remains in teaching AI literacy to optometry students [12].

Optometric education in the UK has moved in recent years from a competency-based educational approach to an outcomes-based approach following the General Optical Council (GOC)’s updated requirements for approved qualifications in optometry in 2021 [13]. In this new approach, the GOC provides high-level outcomes for optometry students to accomplish in the course of their education. While AI is not specifically named in the GOC outcomes for registration, it could be considered that AI technologies fall within outcome 3.3 of the clinical practice category, namely: ‘*Engages with technological advances in eye health and broader healthcare delivery and the significance of specific developments for enhancing patient outcomes and service delivery*’ [13, p. 10]. This high-level outcome allows optometry education providers to have flexibility in how they approach teaching their students about clinical AI and digital technologies. However, this may also lead to ambiguity and optometry education providers may be unclear about what their students actually need to learn about AI applications in optometric practice.

In the field of medical education, much work has been done to understand the attitudes of medical students towards AI, and this has helped to shape the early development of AI modules in the medical curriculum [14–16]. A previous study exploring medical students’ and faculty’s attitudes towards clinical AI found that medical faculty reported greater awareness of AI in medicine than students, but medical faculty were less likely to report a basic understanding of AI than students [17]. However, in the same study, medical students and faculty generally had similar attitudes towards AI applications in medicine. It is not clear how the results of this study, which was conducted at a single medical education institution in 2019, may be comparable with the knowledge levels and attitudes of students and educators at other medical education institutions or across other healthcare disciplines.

While there has been some attempt to explore the attitudes of qualified optometrists towards the use of AI in eye care [18, 19], a comprehensive review of the literature found no previously published survey exploring optometry students’ or educators’ attitudes towards AI in optometry. It is not yet known if there is a difference in knowledge levels or attitudes towards AI in optometric practice between optometry students and educators, or what any difference that exists may be. Therefore, this study intended to address this gap by conducting an online cross-sectional survey, which aimed to answer the following research question: What are the attitudes and knowledge levels of optometry students and educators in the UK regarding the use of AI in optometric practice, and do these differ between the two groups?

## Methods

### Survey Instrument Design

A survey instrument was adapted from a previously published and validated survey by Civaner et al. [14] with the permission of the authors. This survey was chosen from the medical education literature as it explored the perspectives of medical students towards AI use in clinical practice, but also gathered additional information about the educational needs of students to gain clinical AI literacy.

The survey form from Civaner et al. [14] was modified to increase its relevance to optometry students and educators. These modifications included adding questions about the technological savviness of participants and where participants had heard about AI. Questions were derived from a survey of medical students and faculty by Wood et al. [17]. The question ‘*When choosing a field of specialisation, is your choice affected by how artificial intelligence is used in that field?’* [14, p. 4] was removed as it was deemed not to be relevant to optometry students. Likert scale questions were framed in relation to a specific optometry practice scenario: ‘*Imagine you are an optometrist working in practice. Your practice manager has installed a new AI-powered clinical investigation tool. This tool analyses patients’ test results and provides additional information to support your clinical decision-making. Depending on your area of specialty, this tool could be a biometer, topographer, fundus camera or Optical Coherence Tomographer (OCT). Please select to what extent (how much) you agree with the following statements in relation to the optometry practice scenario described above’* (see supplementary material for full survey instrument). Likert scale questions wording was modified to increase their relevance to optometry practice; for example, ‘*AI devalues the medical profession*’ [14, p. 4] became ‘*AI will devalue the optometry profession*’. The list of AI topics to potentially include within the optometry curriculum was developed based on previously published literature about eye care practitioners’ competency requirements regarding the use of AI tools in clinical optometric practice [19–21].

A draft survey was initially piloted on 18 optometry students recruited through convenience sampling from students at the University of Huddersfield. Feedback from the initial pilot participants was gathered and integrated into the survey form prior to wider administration. The only major feedback point from pilot participants was a request for the addition of examples of AI tools for use in optometry. Examples of AI applications for diabetic retinopathy detection, optic disc analysis and an AI patient chatbot were added to the beginning of the survey.

The final survey (included as supplementary material) was structured into four sections:

1. Section 1 gathered information on participant demographic characteristics such as university, age, gender, years of experience and whether the participant was a student or educator.
2. Section 2 contained multiple-choice and multiple-response questions establishing participants’ current knowledge levels and training experience in AI.
3. Section 3 presented a clinical scenario about using an AI-powered clinical investigation tool in optometric practice and required participants to rate their agreement with statements about competence levels using AI tools, negative statements towards AI and positive statements towards AI on a 5-point Likert scale from ‘Strongly Disagree’ to ‘Strongly Agree’.
4. Section 4 required participants to rate AI topics to potentially be included in the optometry curriculum on a 5-point Likert scale from ‘Not Needed’ to ‘Definitely Should Be Included’. An open-ended question allowed participants to suggest their own AI topics for inclusion in the optometry curriculum.

### Survey Administration

The survey was delivered online via Qualtrics (Qualtrics.com) from January to June 2025. The survey was distributed to optometry students and educators across the UK through university optometry student societies, word of mouth, social media and the professional networks of the researchers. The participants recruited through these methods can be considered a convenience sample of voluntary participants. Participant responses were anonymous, and confidentiality was upheld throughout data collection, analysis and interpretation. Informed consent to participate in the study and publish anonymised data from the study was gained from all participants. Participants received no financial remuneration for completing the survey.

### Sample Size Estimate

There are an estimated 3450 optometry students and 200 educators at 15 universities in the UK [22]. Therefore, a minimum sample size of 94 students and 65 educators was calculated to provide 95% certainty and a 10% margin of error [23]. These sample sizes represented target minimum response rates of 2.7% of optometry students and 32.5% of optometry educators in the UK. These figures do not include pre-registration optometrists, who were not considered as students for the purposes of this study and were not invited to participate.

### Statistical Analysis

Descriptive statistics were produced from survey responses. Categorical data were described by frequencies, and Likert-scale data were described using means and standard deviations. Comparisons of responses between students and educators and between genders were performed with the chi-square test for categorical questions and the Mann–Whitney *U* test for Likert-scale questions. As a disproportionate number of responses were received from a single university, a post-hoc subgroup analysis was conducted to examine whether the over-representation of participants from a single institution influenced the results. This subgroup analysis compared the responses of students and educators from the most responsive institution to those from other institutions using the chi-square test for categorical questions and the Mann–Whitney *U* test for Likert-scale questions.

Comparisons across multiple groups, such as age or self-reported knowledge levels about AI, were made with Spearman’s rank correlation. For this analysis, self-reported knowledge levels about AI in optometry were transformed into an ordinal variable on a 5-point scale with 1 being ‘*I have no knowledge at all*’ and 5 being ‘*I am very knowledgeable’*. For the purposes of analysis, training experience was transformed into a binary variable: training or no training.

All statistical analyses were performed using IBM SPSS Statistics for Windows, Version 29.0 (ibm.com). Generally, *p*-values < 0.05 were considered to be statistically significant. For questions 10–12, the Bonferroni correction was applied to adjust for a Type I error. For those questions, *p* < 0.008 (Q10, sources of information about AI), *p* < 0.03 (Q11.1 and Q11.2, competence using AI tools in an optometry clinical practice scenario), *p* < 0.01 (Q11.3–11.7, negative statements towards AI in optometry), *p* < 0.01 (Q11.8–11.12, positive statements towards AI in optometry) and *p* < 0.007 (Q12, AI topics for inclusion in the optometry curriculum) were considered to be statistically significant after the Bonferroni correction was applied. Open-ended questions were inductively coded, and the frequency of codes was reported.

The reliability of the items in Sections 3 and 4 of the survey was calculated using Cronbach’s alpha. It was not possible to conduct test-retest reliability as survey responses were anonymous.

## Results

### Response Rate

A total of 254 responses were received from 213 students and 41 educators. This represented an estimated response rate of 6.2% of optometry students and 20.5% of optometry educators in the UK. For optometry students, this sample size allowed for 95% confidence in the results with a 7% margin of error. Optometry educators did not achieve the minimum target sample size of 65 educators and therefore cannot be said to have achieved 95% confidence with a ≤10% margin of error. A further 90 incomplete responses were received, which were discarded prior to data analysis.

### Respondent Characteristics

Responses were received from 14 out of 15 optometry universities in the UK, with the University of Huddersfield being the most frequent institution. The mean (SD, range) age of respondents was 20.9 (3.1, 18–43) years for students and 41.5 (10.8, 24–76) years for educators. Female respondents accounted for 70.9% (151/213) of students and 68.3% (28/41) of educators. The mean (SD) years of experience for educators was 12.7 (10.7) years.

Table 1 summarises respondent characteristics from Section 1 of the questionnaire.

**Table 1:** Respondent characteristics.

Characteristic	Student respondents *n* (%)	Educator respondents *n* (%)
*Gender*		
Female	151 (70.9)	28 (68.3)
Male	60 (28.2)	13 (31.7)
Non-binary	0 (0.0)	0 (0.0)
Prefer not to say	2 (0.9)	0 (0.0)
*Age (years)*		
18–21	164 (77.0)	0 (0.0)
22–25	37 (17.4)	2 (4.9)
26–45	12 (5.6)	29 (70.7)
46–65	0 (0.0)	9 (21.6)
66 or more	0 (0.0)	1 (2.4)
*University*		
Huddersfield	167 (78.4)	10 (24.4)
Glasgow Caledonian	13 (6.1)	5 (12.2)
Ulster	11 (5.2)	2 (4.9)
Cardiff	7 (3.3)	5 (12.2)
Highlands and Islands	5 (2.3)	0 (0.0)
Teesside	3 (1.4)	2 (4.9)
Hertfordshire	2 (0.9)	3 (7.3)
Aston	2 (0.9)	2 (4.9)
City St George’s	2 (0.9)	1 (2.4)
West of England, Bristol	1 (0.5)	0 (0.0)
Bradford	0 (0.0)	6 (14.6)
Plymouth	0 (0.0)	3 (7.3)
Anglia Ruskin	0 (0.0)	1 (2.4)
Manchester	0 (0.0)	1 (2.4)
*Year of training (students only)*		
1	89 (41.8)	
2	58 (27.2)	
3	58 (27.2)	
4	7 (3.3)	
5	1 (0.5)	
*Years of experience (educators only)*		
1–5		15 (36.6)
6–10		6 (14.6)
11–15		7 (17.1)
16–20		5 (12.2)
21 or more		8 (19.5)

### AI Knowledge Levels and Training Experience

Almost all students (96.2%, 205/213) and more than half of the educators (65.9%, 27/41) had not previously received any training in AI. The difference in training experience between students and educators was statistically significant (*χ*^2^(1) = 40.12, *p* < 0.001, *φ* = 0.40). Most students considered themselves to have either no knowledge at all (35.2%, 75/213) or minimal knowledge (40.4%, 86/213) about AI. Educators were more likely to report they had some knowledge (41.5%, 17/41). The difference in self-reported knowledge levels between students and educators was statistically significant (*χ*^2^(4) = 26.06, *p* < 0.001, *V* = 0.32). Across all participants, those who had undergone AI training reported higher knowledge levels about AI in optometry than those who had not (*U* = 919.00, *p* < 0.001, *r* = 0.33). There were also weak but statistically significant correlations between increasing age and increased knowledge levels about AI in optometry (*r*_s_ = 0.260, *p* < 0.001) and training experience (*r*_s_ = 0.274, *p* < 0.001). There was no statistically significant difference in knowledge levels or training experience by gender. Subgroup analysis for institutional dominance found no statistically significant difference in knowledge levels or training experience of students and educators between those at the most responsive institution and those at other universities.

Table 2 summarises responses from Section 2 of the survey.

**Table 2:**
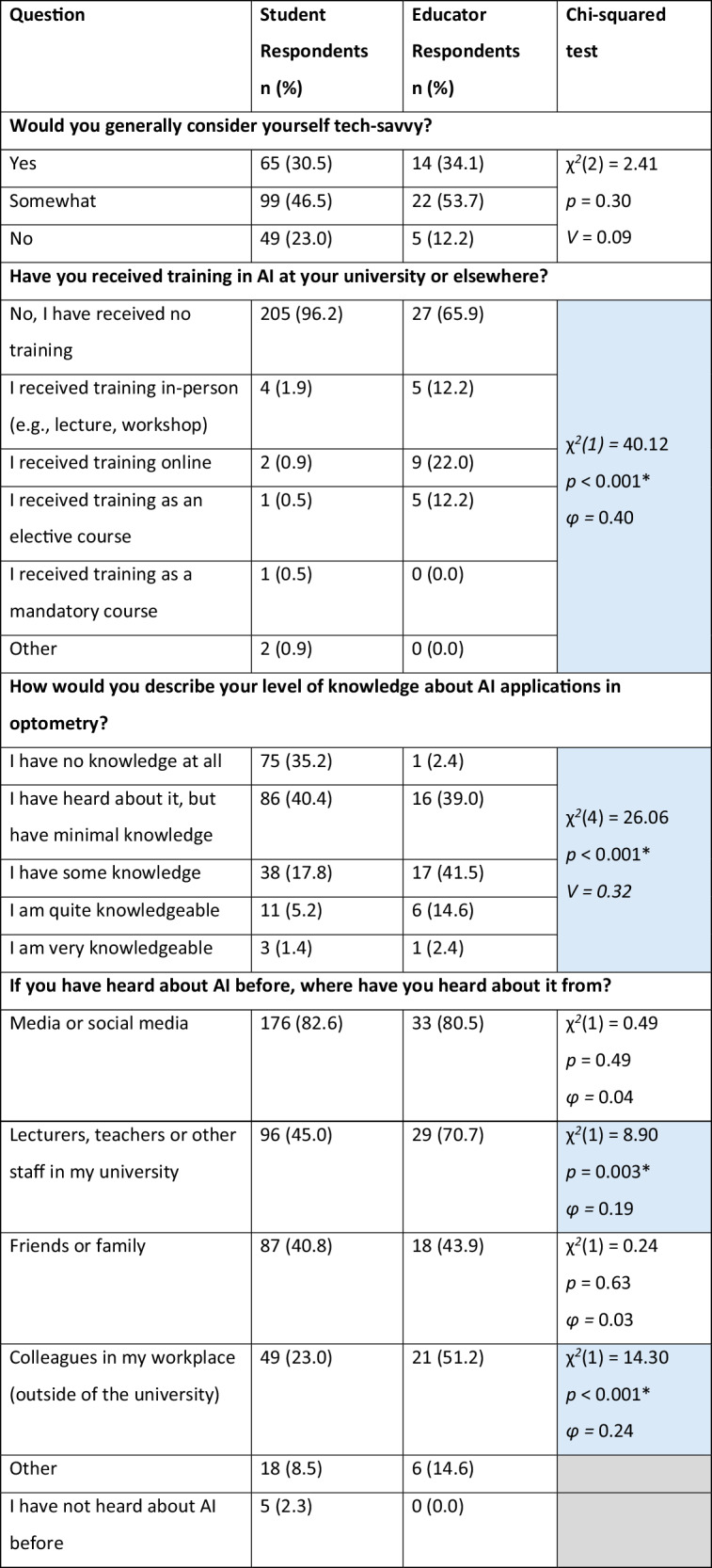
Knowledge levels and training experience in artificial intelligence (AI).

* and a shaded box denotes a statistically significant difference between students and educators.

### Attitudes Towards AI in Optometry

#### Competence Levels Using AI in Optometric Practice

Students generally disagreed with the statement ‘*Currently I feel competent enough to understand how to use AI tools in clinical optometry practice*’, with a mean score (SD) of 2.6 (1.2). Educators were more neutral about this statement with a mean score (SD) of 3.1 (1.1), a difference that was statistically significant (*U* = 3299.00,* p* = 0.01,* r* = 0.16). For the statements regarding competence levels using AI in optometry practice, the Bonferroni correction was applied and a *p*-value < 0.025 was deemed to be statistically significant. Male participants reported they felt more competent than females (*U* = 5132.50, *p* = 0.006,* r* = 0.17). There was a statistically significant correlation between reported competence in understanding how to use AI tools in optometry practice and increasing age; however, this correlation was very weak (*r*_s_ = 0.168,* p* = 0.007). Both students and educators were overall neutral about the statement ‘*Currently I feel competent enough to understand the risks that may occur when using AI tools in clinical optometry practice*’, with a mean score (SD) of 3.1 (1.1) for students and 3.2 (1.3) for educators.

As self-reported knowledge levels about AI in optometry increased, there were statistically significant correlations between competence in understanding how to use AI tools (*r*_s_ = 0.30*, p* < 0.001) and understanding the risks involved in using AI tools (*r*_s_ = 0.215,* p* < 0.001) in optometry practice, although these correlations were weak. Subgroup analysis for institutional dominance found no statistically significant difference in competence levels. Cronbach’s alpha for the questions in this section was 0.674.

#### Negative Statements About AI in Optometry

Educators disagreed significantly more with three out of five negative statements about AI in optometry than students (Table 3). For the negative statements towards AI in optometry, the Bonferroni correction was applied, and a *p*-value < 0.01 was deemed to be statistically significant. The greatest difference in agreement levels was received for the statement ‘*The use of AI in optometry will reduce the need for optometrists and therefore reduce employment opportunities*’, where 34.8% of students agreed with this statement, with a mean score (SD) of 2.9 (1.2), but only 9.8% of educators agreed with a mean score of 2.2 (1.1).

**Table 3:**
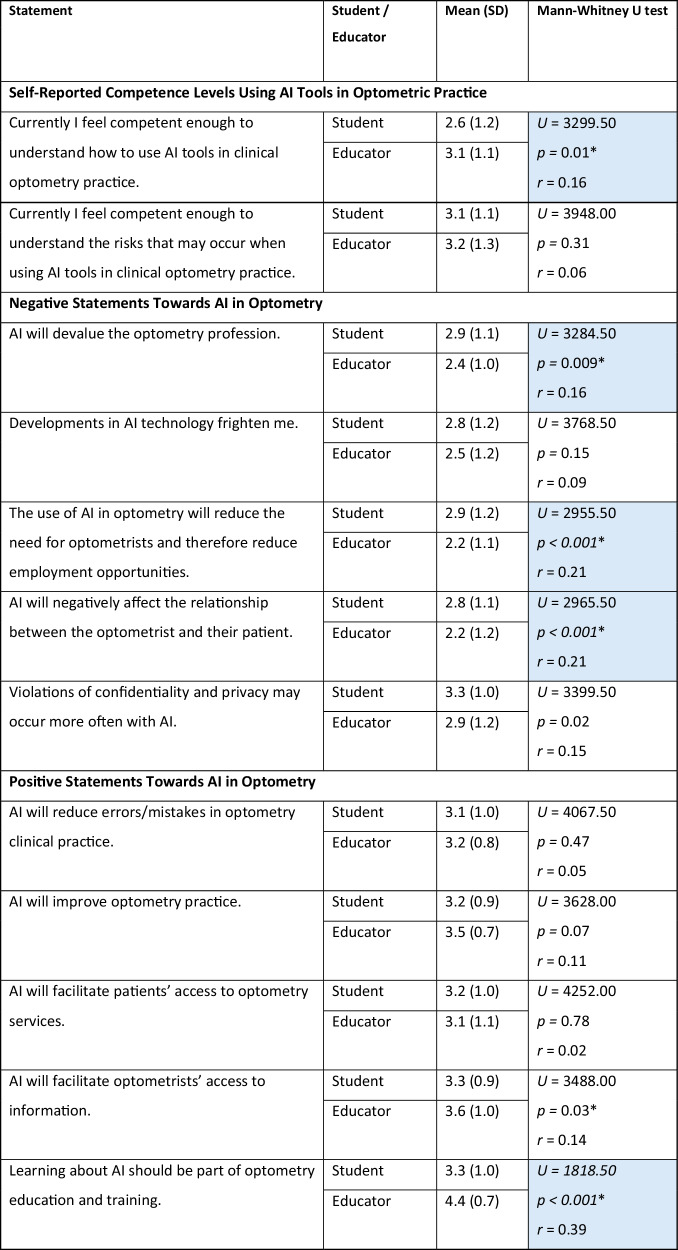
Levels of agreement with statements about competence levels using artificial intelligence (AI) tools in a clinical scenario, and positive and negative statements about AI.

* and shaded box denotes a statistically significant difference between students and educators.

As self-reported knowledge levels about AI in optometry increased, there was a weak but statistically significant negative correlation with levels of agreement for the statement ‘*AI will negatively affect the relationship between the optometrist and their patient*’ (*r*_s_ = −0.218, *p* < 0.001). There was no statistically significant correlation between self-reported AI knowledge levels and the level of agreement with any of the other negative statements.

Educators at the most responsive institution were more likely to disagree with the statement ‘*The use of AI in optometry will reduce the need for optometrists and therefore reduce employment opportunities’* (*U* = 86.00,* p* = 0.03*, r* = 0.34). Subgroup analysis for institutional dominance found no statistically significant difference in levels of agreement with any of the negative statements in students. Cronbach’s alpha for the questions in this section was 0.786.

#### Positive Statements About AI in Optometry

There was no statistically significant difference between educators’ and students’ agreement levels for four out of five positive statements about AI (Table 3). However, educators agreed significantly more strongly than students that ‘*Learning about AI should be part of optometry education and training*’, with a mean score (SD) of 4.4 (0.7) for educators and 3.3 (1.0) for students (*U* = 1818.50, *p* < 0.001, *r* = 0.39). For the positive statements towards AI in optometry, the Bonferroni correction was applied, and a *p*-value < 0.01 was deemed to be statistically significant.

As knowledge levels about AI increased, there were weak but statistically significant correlations with levels of agreement for the statements ‘A*I will improve optometry practice*’ (*r*_s_ = 0.228, *p* < 0.001) and ‘*Learning about AI should be part of optometry education and training*’ (*r*_s_ = 0.303, *p* < 0.001). There was no statistically significant correlation between self-reported AI knowledge levels and the level of agreement with any of the other positive statements.

There was a statistically significant difference in attitudes towards all but one of the statements in Section 3 between those participants who had undergone AI training and those who had not. The only statement that did not have any significant difference in attitudes between those who had and had not received AI training was ‘*Violations of confidentiality and privacy may occur more often with AI*’ (*U* = 2529.00, *p* = 0.94, *r* = 0.004).

Students at the most responsive institution were more likely to disagree with the statement ‘*Learning about AI should be part of optometry education and training’* (*U* = 2898.00, *p* = 0.008, *r* = 0.18). Subgroup analysis for institutional dominance found no statistically significant difference in levels of agreement with positive statements for educators. Cronbach’s alpha for the questions in this section was 0.755.

### AI Learning Priorities for Optometry Students

Educators were in general agreement that all of the proposed topics should be included in the optometry curriculum, while students felt more neutral about the proposed AI topics.

‘*How to interpret the results/outputs of AI tools*’ was the most important topic to include in the curriculum according to educators, with a mean score (SD) of 4.6 (0.7), although many other topics such as ‘*How to appraise the accuracy and reliability of an AI tool*’ and ‘*Regulations and legal frameworks surrounding the use of AI tools in clinical practice*’ received similarly high scores from educators, each with a mean score (SD) of 4.5 (0.6).

Students ranked the topics ‘*How to interpret the results/outputs of AI tools’, ‘What AI tools are available for use by optometrists*’, ‘*Training to prevent and solve ethical problems that may arise when using AI tools*’ and ‘*Regulations and legal frameworks surrounding the use of AI tools in clinical practice*’ highest, each with a mean score (SD) of 3.8 (1.0). The least important topic for both students and educators was ‘*How AI tools are developed, trained and validated*’ with a mean score (SD) of 3.1 (1.1) and 3.8 (1.2), respectively.

Students at the most responsive institution were more likely to disagree that five out of the seven presented topics should be included in the optometry curriculum, which was consistent with their lower agreement with the statement ‘*Learning about AI should be part of optometry education and training’* in the previous section. Subgroup analysis for institutional dominance found no statistically significant difference in responses for educators. Cronbach’s alpha for the questions in this section was 0.890.

Table 4 summarises responses from Section 4 of the survey.

**Table 4:**
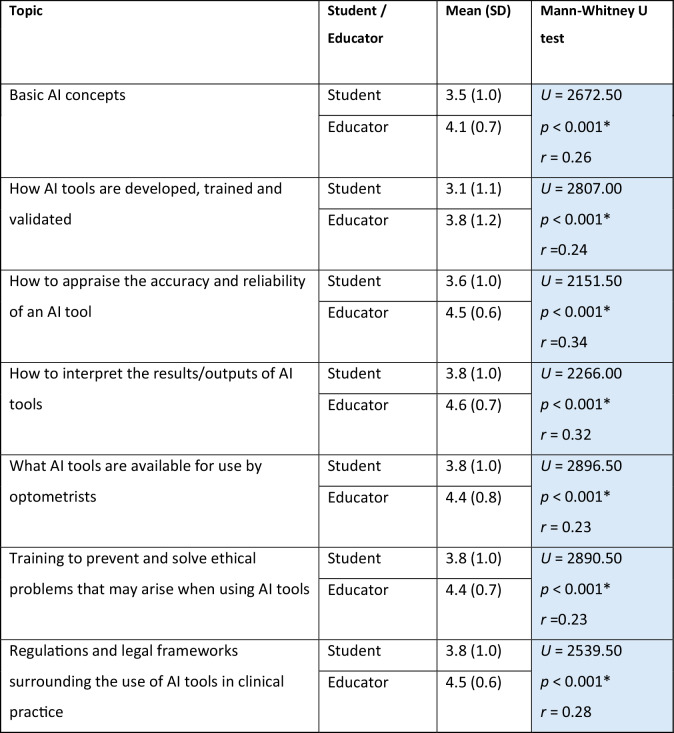
Artificial intelligence (AI) learning priorities for the optometry curriculum.

* and shaded box denotes statistically significant difference between students and educators.

An open-ended question received the following suggestions for additional topics of importance from students:

- Effective communication with AI and knowing how to input information to receive the best possible results (*n* = 3).
- AI use in ocular disease screening and diagnosis (*n* = 2).
- How to use AI to aid referrals between primary and secondary eye care (*n* = 1).
- How to use AI to collaborate with other health professionals (*n* = 1).
- How AI can reduce waiting times (*n* = 1).
- Knowing when to use AI and when not to (*n* = 1).
- Using AI to spot anything small that could be missed by human practitioners (*n* = 1).
- How AI could be used in learning/education (*n* = 1).

Educators suggested the following additional topics of importance:

- Professional uses of AI, for example, writing documents (*n* = 1).
- The risks associated with AI use (*n* = 1).
- How to inform patients and address patient concerns regarding AI use (*n* = 1).
- Staff need more training on it, and universities need to have a robust and definitive policy regarding AI (*n* = 1).
- Students need to know what the university and GOC stance is on AI and in using it for clinics, and also in generating work for projects/dissertations (*n* = 1).

The topics above represent a complete list of all responses received.

## Discussion

This study investigated the perspectives of optometry students and educators in the UK on the use of AI in optometric practice through an online cross-sectional survey. The authors believe this to be the first survey of its kind specifically exploring the views of optometry students and educators towards AI in optometric practice. The responses indicated that optometry students currently have very limited knowledge levels about AI in optometric practice. Students were less likely to have undergone AI training and more likely to hold negative attitudes towards AI in optometry than educators. Across all respondents, previous AI training was associated with greater self-reported confidence using AI tools in optometry practice, greater agreement with positive statements about AI applications in optometry and greater disagreement with negative statements about AI applications in optometry. Educators felt strongly that teaching about AI should be included in the optometry curriculum. Potential AI topics for inclusion in the optometry curriculum were explored.

Almost all of the optometry students surveyed (96.2%) reported they had not received any training on AI. While there is no previously published survey with optometry students for comparison, when considering the literature from medical education, these results are similar to those of Sit et al. [24] and Laupichler et al. [25], who found that 90.8 and 91.0% of medical students, respectively, had not received any AI training. The present results, as in both Sit et al. [24] and Laupichler et al. [25], found that those who had undergone training were more likely to report higher knowledge and competence levels in using AI tools, highlighting that AI training may play an important role in helping health professions students gain AI literacy.

When exploring attitudes towards AI more generally, it has been found that people aged 20–35 years have more positive attitudes towards AI than those aged 40–50 years [26]. In contrast, the current results, which explored attitudes specifically towards AI in optometric practice, found that educators were more knowledgeable and less negative towards AI in optometry than students. A recent survey by the College of Optometrists in the UK discovered that younger optometrists were more sceptical about AI than older practitioners [27]. This may indicate that differences in attitudes towards AI in optometry exist between different age groups, although more research is required to explore this effect in greater detail. Nonetheless, it is important for optometry educators to avoid assuming that students will be interested in AI or have a high baseline level of knowledge about AI when developing and delivering future curricula.

An additional consideration when implementing AI curricula within optometry programmes is whether optometry educators have the required expertise about AI in order to teach the subject. While educators were generally more knowledgeable about AI applications in optometry than the students in this study, only one of the educator participants reported that they were ‘*very knowledgeable’*. Similarly, lack of faculty expertise in AI has been cited as a significant challenge to integrating AI literacy into the medical curriculum [28] and more broadly across health professions education [29]. It has been proposed that interdisciplinary and interfaculty collaborations could help to overcome this challenge [30]. However, it is likely that faculty development and training initiatives for optometry educators will be required prior to the implementation of AI literacy training for optometry students, as has been recommended for nursing and other health professions [29]. Limited educator knowledge levels about AI are just one of several potential barriers to integrating AI literacy into the optometry curriculum. Further research is required to explore the institutional readiness of optometry education providers to provide AI training to their optometry students.

It is also important to consider the impact of gender. Blease et al. [31] found that male medical students had greater self-reported awareness of clinical AI and were more likely to plan to learn about AI than female medical students. While the present study found no difference in AI knowledge levels by gender, it was noted that males were more likely to self-report competence using AI tools in clinical practice compared with females. However, the size of this effect was small. Furthermore, it has been shown previously among health professions students that males frequently have higher self-reported confidence scores in clinical skills, knowledge and other competencies, even when no objective difference in competency levels actually exists [32]. Therefore, it is important to avoid reinforcing gender stereotypes by assuming that male optometry students may be more proficient in technological subjects such as AI compared with female students, when the evidence may not support this assumption.

Finally, a further consideration when developing an AI curriculum for optometry students is what content to teach. In the UK, the GOC’s outcomes-based approach states that technological advancements in eye care delivery should be covered by optometry educators [13], but specific topics about technological advancements, such as clinical AI, are not specified and are left for optometry education providers themselves to determine. This study asked participants to rate AI topics by importance for inclusion in a future curriculum, and found that educators were generally enthusiastic about including all proposed topics, while students felt more neutral. However, both students and educators rated ‘*How AI tools are developed, trained and validated*’ as the least important topic to include in the optometry curriculum. In contrast, Murphy et al. [20] and Aslam and Hoyle [21] both focused on knowledge of AI training and development as being important to understand in order for eye care professionals to integrate AI tools into their clinical decision-making processes.

Furthermore, the recently published ‘Interim position on the use of AI in eye care’ from the UK College of Optometrists recommends that optometrists understand the training datasets and validation methods of AI diagnostic tools prior to their procurement and implementation [33]. However, as the participants in this study generally had low levels of knowledge about AI, the task of rating the importance of AI topics may have been challenging, as it is difficult to appreciate what is useful to know about a subject if one’s own knowledge of the subject is minimal. Therefore, the results of this study represent an early exploration of potential AI topics to include in optometry curricula. Further research is required to determine what UK optometry students should be learning about AI applications in optometry, particularly as it relates to accomplishing the GOC’s required outcome for understanding technological advancements in eye health and broader healthcare delivery [13].

## Limitations

It is important to consider the findings of this study within the context of its limitations.

The majority of student responses (78.4%) were received from one institution. Subgroup analyses were performed to compare the responses of students and educators from the most responsive institution with those from all other universities. These analyses showed that institutional dominance in the responses did not meaningfully influence the main findings. Differences found were limited to a small number of items and did not alter the overall conclusions of this study. Additionally, the demographics of the student participants in this study overall were broadly similar to the GOC published data on optometry student demographics in the UK by age and gender [34]. However, the participants of this study still should not be considered a nationally representative sample of students.

The number of educator responses to this survey was less than the target response rate for a 95% confidence level with a ≤10% margin of error, which limits the reliability of the educator results. As such, these results should be interpreted with caution.

The survey was conducted on a voluntary basis, which could lead to self-selection bias and potentially only students with a prior interest in AI completing the survey. As the survey was conducted online, responses may over-represent digitally engaged participants. However, the student responses received by this survey were comparable with other similar surveys on other health professional students in the literature, indicating that this, as a source of bias, is likely to be limited.

The survey was designed to take no longer than 5–10 min to complete, and the number of questions was limited to avoid non-response bias and neutral or extreme response bias due to boredom or fatigue. However, in order to limit the number of questions included and prevent an overly long survey, no reverse-worded questions were included to check for acquiescence bias. Additionally, while the modified survey instrument was developed by a team of experienced optometric educators and has undergone face validity through expert opinion in this context, the content validity of the modified survey was not reassessed formally, which could be considered a limitation.

Furthermore, the survey explored self-reported knowledge levels and attitudes towards AI in optometric practice only and did not conduct any objective measures of clinical AI literacy or competence.

Finally, this survey was distributed to UK-based optometry students and educators only, and therefore may not be generalisable to other locations or contexts.

## Conclusion

Optometry students’ current self-reported knowledge levels about AI applications in optometry are very limited. While educators were found to have higher self-reported knowledge levels about AI in optometry than students, knowledge about AI among educators could still be considered somewhat limited, which may be a barrier to implementing AI training for optometry students in the university curriculum. A wide range of AI topics were found to be potentially suitable to teach to optometry students. This research provides an early exploration of the considerations for creating future AI educational initiatives in optometry programmes; however, the present cross-sectional study had several methodological limitations. Therefore, further research in this area is required; in particular, a need for objective assessments of AI literacy among optometry students and educators and for multi-institutional, longitudinal studies.

## Supplementary Information

Resubmission - AI Survey (Appendix)

## Data Availability

No datasets were generated or analysed during the current study.
