# Supplementary material for: Attitudes and Knowledge Levels of Optometry Students and Educators Towards Artificial Intelligence in Optometric Practice: An Online Cross-Sectional Survey
Source: Ophthalmic Physiol Opt. 2026 Mar 6;46(2):419–28. doi: 10.1007/s44402-026-00053-z (PMC13369741; doi:10.1007/s44402-026-00053-z)
Supplement: Supplementary file 1 — Resubmission - AI Survey (Appendix) [file 44402_2026_53_MOESM1_ESM.docx]

## Survey Participant Information Sheet

**Study Title:** The integration of artificial intelligence in optometry education.

**Introduction:**

You are being invited to participate in a research study which aims to explore the perspectives of optometry students and educators about the use of artificial intelligence (AI) in optometry education and clinical practice.

This study is being conducted by [*name redacted*] as part of her PhD research at the University of Huddersfield with supervision provided by [*name redacted*], [*name redacted*] and [*name redacted*].

Before you decide to participate, it is important for you to understand what participation in the study will involve. Please read the following information sheet carefully. You may discuss it with others if you wish. Contact details are provided at the end of this form if you have further questions or require any further information.

**Why is this study important?**

This is an important study as it will allow for key stakeholder views from optometry students and educators to inform the development of an optometric education curriculum which better prepares student optometrists for the AI-powered optometry practice of the future.

**Why have I been invited to participate?**

We believe that your perspective as either an optometry student or educator will allow us to gain a greater understanding of the how to improve the AI knowledge and skills of optometry students and how to integrate AI literacy into the optometry curriculum.

**What will happen if I agree to take part?**

You will be invited to participate in an online survey where you will be asked a series of questions regarding your perspectives on the use of AI in optometry practice, your experience of AI training so far (if any), and your perspectives on potential AI topics for inclusion in a future optometry curriculum.

The survey is expected to take no more than 10 minutes to complete.

Following submission of the survey, if you wish to amend any response, you may contact the lead researcher [*name redacted*] *[email address redacted*]*.*

**Do I have to take part?**

Participation is voluntary. You are free to withdraw from the study at any time, without giving any reason. Withdrawing from the study will not affect your legal rights. If you withdraw from the study, any data collected prior to your withdrawal which has not yet been anonymised will be destroyed and will not be included in any subsequent reports or publications. It is not possible to withdraw and destroy any data which has already been anonymised. To withdraw from the study, email the lead researcher [*name redacted*] *[email address redacted*].

**What are the possible risks involved in taking part?**

All studies involve some level of risk or inconvenience. There may be possible risks associated with data protection and privacy. There are many procedures in place to minimise these risks and ensure the secure storage of the data.

**What are the possible benefits of taking part?**

Whilst participating in this study may not directly benefit you personally, by taking part in this study you will contribute to the development of a new optometry curriculum which better prepares students for the AI-powered optometry practice of the near future. There is no remuneration for taking part in this study.

**What will happen to the data gathered during this study?**

Personal data that will be collected may include age, gender, nationality, occupation, education level, and university of attendance/employment. All data collected will be stored in a secure encrypted cloud-based storage folder which only the researchers on this project will have access to. Data will be destroyed confidentially 5 years after successful completion of the lead researcher’s PhD.

This study is compliant with the Data Protection Act (DPA) (2018) and the General Data Protection Regulation (GDPR) (EU) (2016/679). The data controller is the University of Huddersfield. Data is processed under the basis of Article 6(1)(e) of GDPR to perform a task carried out in the public interest.

Any questions or concerns relating specifically to data protection can be made to the University of Huddersfield’s Data Protection Officer (data.protection@hud.ac.uk). If you are unhappy with the response from the University of Huddersfield, you have the right to lodge a complaint with the Information Commissioner’s Office (casework@ico.org.uk).

GDPR (2016/679) also gives study participants the right to ask for their personal data to be erased. If you would like us to stop using your personal data, contact the lead researcher (Fiona Buckmaster). However, it will only be possible to erase data that has not been anonymised and/or published. Further information about your rights can be found at: https://www.hud.ac.uk/informationgovernance/dataprotection/.

**What will happen to the results of this study?**

The results of this study will be used to inform the development of an ‘AI for optometrists’ seminar for optometry students. Ultimately, the results of this study will be used to inform a framework for fully integrating AI literacy into the optometry university curriculum. The findings of this study and subsequent curriculum framework will be included in a PhD thesis, and may be published in academic journals, conference presentations or posters, or used for educational purposes.

A summary of the findings of this study can be provided to you upon request from [*name redacted*] *[email address redacted*].

**What if I have a problem?**

If you have any questions or concerns, you are welcome to contact the members of the research team at any time. However, if you are concerned about your participation in this study and would like to speak to someone outside of the study team, please contact [*name redacted*] *[contact details redacted*].

**Does this study have ethical approval?**

This study was approved by the University of Huddersfield’s School of Applied Sciences Research Ethics and Integrity Committee, reference SAS-SRIEC-FB.10.12.2024.

**Who can I contact for more information?**

If you have any further questions about this study, please contact:

Lead Researcher: [*name redacted*] *[email address redacted*]

Principal Supervisor: [*name redacted*] *[email address redacted*]

**Thank you for taking the time to read this information and for considering participating in this study.**

**AI in Optometry Example – Your MD:**


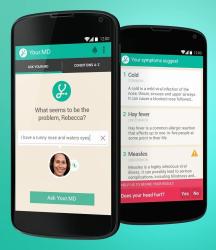

**AI Medical Chatbot.**
Image credit: <https://ribot.co.uk/work/your-md>

**AI in Optometry Example – Diabetic Retinopathy:**


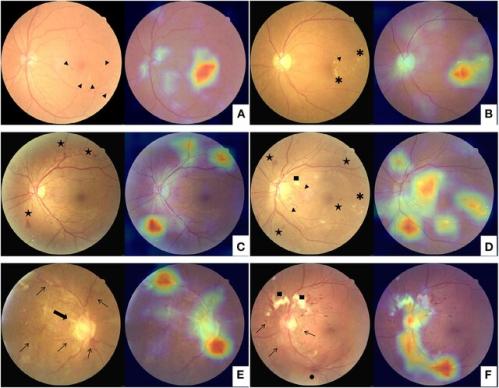

**AI analysis of diabetic retinopathy in fundus photographs.**
Image credit: Qian, X., et al. (2022) 'The effectiveness of artificial intelligence-based automated grading and training system in education of manual detection of diabetic retinopathy.' Frontiers in Public Health, 10:1025271.

**AI in Optometry Example – Optic Disc Analysis:**


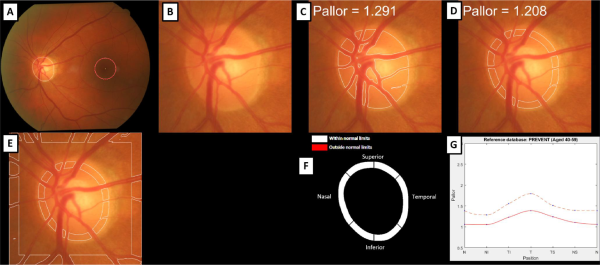

**AI analysis of optic disc pallor.**
Image credit: Gibbon, S., et al. (2024) 'PallorMetrics: Software for Automatically Quantifying Optic Disc Pallor in Fundus Photographs, and Associations with Peripapillary RNFL Thickness.' Trans Vis Sci, 13(5), 20.

## Survey Instrument

**1. University:** _______________________________________________________________

**2. Are you a:** □ Student □ Educator

*If answered Q2 as “Student”:*

**3. Age:** ________

**4. Gender:** □ Female □ Male □ Non-binary □ Prefer not to say

**5. Nationality:** ______________________________

**6. Year of training:** 1 2 3 4 5

**7. Would you generally consider yourself “tech savvy”?**

□ Yes □ No □ Somewhat

**8. Have you received training in AI at your university or elsewhere?** *(select all that apply)*

1. No, I have received no training.
2. I received training as an elective course.
3. I received training as a mandatory course.
4. I received training in-person (e.g. lecture, workshop).
5. I received training online.
6. Other: _____________________

**9. How would you describe your level of knowledge about AI applications in optometry?**

1. I have no knowledge at all.
2. I have heard about it, but have minimal knowledge.
3. I have some knowledge.
4. I am quite knowledgeable.
5. I am very knowledgeable.

**10. If you have heard about AI before, where have you heard about it from?** *(select all that apply)*

1. Media or social media
2. Lecturers, teachers, or other staff in my university
3. Colleagues in my workplace (outside of the university)
4. Friends or family
5. Other: _____________________
6. I have not heard about AI before

**11. Imagine you are an optometrist working in practice. Your practice manager has installed a new AI-powered clinical investigation tool. This tool analyses patients’ test results and provides additional information to support your clinical decision making. Depending on your area of specialty, this tool could be a biometer, topographer, fundus camera or OCT.**

**Please select to which extent (how much) you agree with the following statements in relation to the optometry practice scenario described above:**

| **Statement** | **Strongly agree** | **Somewhat agree** | **Neither agree nor disagree** | **Somewhat disagree** | **Strongly disagree** |
| --- | --- | --- | --- | --- | --- |
| Currently I feel competent enough to understand how to use AI tools in clinical optometry practice. | 5 | 4 | 3 | 2 | 1 |
| Currently I feel competent enough to understand the risks that may occur when using AI tools in clinical optometry practice. | 5 | 4 | 3 | 2 | 1 |
| AI will devalue the optometry profession. | 5 | 4 | 3 | 2 | 1 |
| Developments in AI technology frighten me. | 5 | 4 | 3 | 2 | 1 |
| The use of AI in optometry will reduce the need for optometrists and therefore reduce employment opportunities. | 5 | 4 | 3 | 2 | 1 |
| AI will negatively affect the relationship between the optometrist and their patient. | 5 | 4 | 3 | 2 | 1 |
| Violations of confidentiality and privacy may occur more often with AI. | 5 | 4 | 3 | 2 | 1 |
| AI will reduce errors/mistakes in optometry clinical practice. | 5 | 4 | 3 | 2 | 1 |
| AI will improve optometry practice. | 5 | 4 | 3 | 2 | 1 |
| AI will facilitate patients’ access to optometry services. | 5 | 4 | 3 | 2 | 1 |
| AI will facilitate optometrists’ access to information. | 5 | 4 | 3 | 2 | 1 |
| Learning about AI should be part of optometry education and training. | 5 | 4 | 3 | 2 | 1 |

**12. Please choose to which degree you think the following topics should be included in the optometry curriculum:**

| **Topic** | **Definitely should be included** | **Would be good** | **Unsure** | **Not a must** | **Not needed** |
| --- | --- | --- | --- | --- | --- |
| Basic AI concepts | 5 | 4 | 3 | 2 | 1 |
| How AI tools are developed, trained and validated | 5 | 4 | 3 | 2 | 1 |
| How to appraise the accuracy and reliability of an AI tool | 5 | 4 | 3 | 2 | 1 |
| How to interpret the results/outputs of AI tools | 5 | 4 | 3 | 2 | 1 |
| What AI tools are available for use by optometrists | 5 | 4 | 3 | 2 | 1 |
| Training to prevent and solve ethical problems that may arise when using AI tools | 5 | 4 | 3 | 2 | 1 |
| Regulations and legal frameworks surrounding the use of AI tools in clinical practice | 5 | 4 | 3 | 2 | 1 |

**13. Any other topics about AI you think should be included in the optometry curriculum:**

_______________________________________________________________________________

**14. In the future, we may conduct online interviews about the use of AI in optometry practice. Please let us know if you are happy to be contacted about possibly taking part in these interviews.**

□ Yes □ No If yes, please provide email address: _________________________

**Thank you for your participation in this study.**

*If answered Q2 as “Educator”:*

**3. Age:** ________

**4. Gender:** □ Female □ Male □ Non-binary □ Prefer not to say

**5. Nationality:** ______________________________

**6. Years working in optometry education: ______________________**

**7. Would you generally consider yourself “tech savvy”?**

□ Yes □ No □ Somewhat

**8. Have you received training in AI at your university or elsewhere?** *(select all that apply)*

1. No, I have received no training.
2. I received training as an elective course.
3. I received training as a mandatory course.
4. I received training in-person (e.g. lecture, workshop).
5. I received training online.
6. Other: _____________________

**9. How would you describe your level of knowledge about AI applications in optometry?**

1. I have no knowledge at all.
2. I have heard about it, but have minimal knowledge.
3. I have some knowledge.
4. I am quite knowledgeable.
5. I am very knowledgeable.

**10. If you have heard about AI before, where have you heard about it from?** *(select all that apply)*

1. Media or social media
2. Lecturers, teachers, or other staff in my university
3. Colleagues in my workplace (outside of the university)
4. Friends or family
5. Other: _____________________
6. I have not heard about AI before

**11. Imagine you are an optometrist working in practice. Your practice manager has installed a new AI-powered clinical investigation tool. This tool analyses patients’ test results and provides additional information to support your clinical decision making. Depending on your area of specialty, this tool could be a biometer, topographer, fundus camera or OCT.**

**Please select to which extent you agree with the following statements in relation to the optometry practice scenario described above:**

| **Statement** | **Strongly agree** | **Somewhat agree** | **Neither agree nor disagree** | **Somewhat disagree** | **Strongly disagree** |
| --- | --- | --- | --- | --- | --- |
| Currently I feel competent enough to understand how to use AI tools in clinical optometry practice. | 5 | 4 | 3 | 2 | 1 |
| Currently I feel competent enough to understand the risks that may occur when using AI tools in clinical optometry practice. | 5 | 4 | 3 | 2 | 1 |
| AI will devalue the optometry profession. | 5 | 4 | 3 | 2 | 1 |
| Developments in AI technology frighten me. | 5 | 4 | 3 | 2 | 1 |
| The use of AI in optometry will reduce the need for optometrists and therefore reduce employment opportunities. | 5 | 4 | 3 | 2 | 1 |
| AI will negatively affect the relationship between the optometrist and their patient. | 5 | 4 | 3 | 2 | 1 |
| Violations of confidentiality and privacy may occur more often with AI. | 5 | 4 | 3 | 2 | 1 |
| AI will reduce errors/mistakes in optometry clinical practice. | 5 | 4 | 3 | 2 | 1 |
| AI will improve optometry practice. | 5 | 4 | 3 | 2 | 1 |
| AI will facilitate patients’ access to optometry services. | 5 | 4 | 3 | 2 | 1 |
| AI will facilitate optometrists’ access to information. | 5 | 4 | 3 | 2 | 1 |
| Learning about AI should be part of optometry education and training. | 5 | 4 | 3 | 2 | 1 |

**12. Please choose to which degree you think the following topics should be included in the optometry curriculum:**

| **Topic** | **Definitely should be included** | **Would be good** | **Unsure** | **Not a must** | **Not needed** |
| --- | --- | --- | --- | --- | --- |
| Basic AI concepts | 5 | 4 | 3 | 2 | 1 |
| How AI tools are developed, trained and validated | 5 | 4 | 3 | 2 | 1 |
| How to appraise the accuracy and reliability of an AI tool | 5 | 4 | 3 | 2 | 1 |
| How to interpret the results/outputs of AI tools | 5 | 4 | 3 | 2 | 1 |
| What AI tools are available for use by optometrists | 5 | 4 | 3 | 2 | 1 |
| Training to prevent and solve ethical problems that may arise when using AI tools | 5 | 4 | 3 | 2 | 1 |
| Regulations and legal frameworks surrounding the use of AI tools in clinical practice | 5 | 4 | 3 | 2 | 1 |

**13. Any other topics about AI you think should be included in the optometry curriculum:**

_______________________________________________________________________________

**14. In the future, we may conduct online interviews about the use of AI in optometry practice. Please let us know if you are happy to be contacted about possibly taking part in these interviews.**

□ Yes □ No If yes, please provide email address: _________________________

**Thank you for your participation in this study.**
